# Notch1 signaling in *NOTCH1*-mutated mantle cell lymphoma depends on *Delta-Like* ligand 4 and is a potential target for specific antibody therapy

**DOI:** 10.1186/s13046-019-1458-7

**Published:** 2019-11-01

**Authors:** Elisabeth Silkenstedt, Fabian Arenas, Berta Colom-Sanmartí, Sílvia Xargay-Torrent, Morihiro Higashi, Ariadna Giró, Vanina Rodriguez, Patricia Fuentes, Walter E. Aulitzky, Heiko van der Kuip, Sílvia Beà, Maria L. Toribio, Elias Campo, Mònica López-Guerra, Dolors Colomer

**Affiliations:** 1 Experimental Therapeutics in Lymphoid Malignancies Group, Institut d’Investigacions Biomèdiques August Pi i Sunyer (IDIBAPS), Barcelona, Spain; 2 Department of Internal Medicine III, University Hospital, Ludwig Maximilian University, Munich, Germany; 3 Dr. Margarete Fischer-Bosch-Institute of Clinical Pharmacology and University of Tuebingen, Stuttgart, Germany; 4 Centro de Investigación Biomédica en Red de Cáncer (CIBERONC), Madrid, Spain; 5 Centro de Biología Molecular Severo Ochoa, Consejo Superior de Investigaciones Científicas, Universidad Autónoma de Madrid, Madrid, Spain; 6 Department of Hematology and Oncology, Robert-Bosch-Hospital, Stuttgart, Germany; 7 Lymphoid Neoplasm Program, IDIBAPS, Barcelona, Spain; 8 Hematopathology Section, Hospital Clínic, Barcelona, Spain; 9 University of Barcelona, Barcelona, Spain

**Keywords:** Mantle cell lymphoma, Notch1, Delta-like ligand, Angiogenesis, OMP-52 M51

## Abstract

**Background:**

*NOTCH1* gene mutations in mantle cell lymphoma (MCL) have been described in about 5–10% of cases and are associated with significantly shorter survival rates. The present study aimed to investigate the biological impact of this mutation in MCL and its potential as a therapeutic target.

**Methods:**

Activation of Notch1 signaling upon ligand-stimulation and inhibitory effects of the monoclonal anti-Notch1 antibody OMP-52M51 in *NOTCH1*-mutated and -unmutated MCL cells were assessed by Western Blot and gene expression profiling. Effects of OMP-52M51 treatment on tumor cell migration and tumor angiogenesis were evaluated with chemotaxis and HUVEC tube formation assays. The expression of *Delta-like ligand 4* (DLL4) in MCL lymph nodes was analyzed by immunofluorescence staining and confocal microscopy. A MCL mouse model was used to assess the activity of OMP-52M51 in vivo.

**Results:**

Notch1 expression can be effectively stimulated in *NOTCH1*-mutated Mino cells by DLL4, whereas in the *NOTCH1-*unmutated cell line JeKo-1, less effect was observed upon any ligand-stimulation. DLL4 was expressed by histiocytes in both, *NOTCH1*-mutated and –unmutated MCL lymph nodes. Treatment of *NOTCH1*-mutated MCL cells with the monoclonal anti-Notch1 antibody OMP-52M51 effectively prevented DLL4-dependent activation of Notch1 and suppressed the induction of numerous direct Notch target genes involved in lymphoid biology, lymphomagenesis and disease progression. Importantly, in lymph nodes from primary MCL cases with *NOTCH1/2* mutations, we detected an upregulation of the same gene sets as observed in DLL4-stimulated Mino cells. Furthermore, DLL4 stimulation of *NOTCH1*-mutated Mino cells enhanced tumor cell migration and angiogenesis, which could be abolished by treatment with OMP-52M51. Importantly, the effects observed were specific for *NOTCH1*-mutated cells as they did not occur in the *NOTCH1*-wt cell line JeKo-1. Finally, we confirmed the potential activity of OMP-52M51 to inhibit DLL4-induced Notch1-Signaling in vivo in a xenograft mouse model of MCL.

**Conclusion:**

DLL4 effectively stimulates Notch1 signaling in *NOTCH1*-mutated MCL and is expressed by the microenvironment in MCL lymph nodes. Our results indicate that specific inhibition of the Notch1-ligand-receptor interaction might provide a therapeutic alternative for a subset of MCL patients.

## Background

Although with current standard therapy high initial response rates can be achieved, early relapses and rapid disease progression determine the clinical course of most mantle cell lymphoma (MCL) patients [1]. In the last years, new therapies were approved targeting the proteasome, the PI3K-Akt-mTOR pathway, the B-cell receptor (BCR) signaling pathway and the anti-apoptotic Bcl-2 protein family. Yet, MCL remains an incurable disease [2]. The t (11;14)(q13;q32) translocation leading to Cyclin D1 overexpression is the primary oncogenic event in MCL pathogenesis [3]. Furthermore, constitutive activation of BCR signaling plays an important role in disease development [4]. Additionally, genomic profiling revealed a high number of secondary genetic alterations and recurrent mutations affecting cell cycle, DNA damage response and apoptosis pathways that contribute to pathogenesis and aggressiveness of MCL [3]. Among them, *NOTCH1* gene mutations have been described with a frequency of 5–10% and were shown to be associated with shorter survival rates [5, 6]. Therefore, further investigation of the biological effect of this mutation in MCL and its potential as a therapeutic target is of great interest.

The majority of the previously described *NOTCH1*-mutations in MCL consist of either small frameshift-causing indels or nonsense mutations in exon 34 [6]. These mutations lead to truncation of the C-terminal PEST-domain, thereby removing the recognition site from the ubiquitin ligase degradation complex, resulting in a more stable and transcriptionally active form of Notch1-intracellular domain (NICD). The Notch signaling pathway operates in a context- and tissue-dependent way participating in diverse cellular processes, such as cancer stem cell biology, angiogenesis, cell proliferation and survival [7]. In addition to the well-known Notch target genes *HES1* and *HEY1*, two basic-helix-loop-helix (Bhlh)-class of transcription factors, numerous genes have been identified as directly regulated by activated Notch1 [8]. Some cancer-related target genes of Notch include NF-κB family members, *CYCLIN D1, p21, GATA3, MYC* and *DTX1* [9]. In mammals, Notch signaling is usually activated upon interactions with Delta-like ligands (DLL1, DLL3, DLL4) and Jagged ligands (JAG1, JAG2), resulting in a series of proteolytic cleavage events that finally release NICD from its membrane receptor and lead to its nuclear translocation [7].

Targeting Notch signaling has been studied in various cancer types and particularly using gamma-secretase inhibitors (GSI) in hematological malignancies [6, 10, 11]. However, the clinical applicability of GSI is limited as it can cause severe diarrhea resulting from simultaneous inhibition of Notch1 and Notch2 signaling in gut epithelial stem cells [12, 13]. Thus, alternative strategies for therapeutic targeting of Notch1 are highly warranted. Recently, antibodies that inhibit signaling of both, wild-type and mutated Notch1 receptors have been characterized [14]. OMP-52M51 (brontictuzumab) is a full length IgG2 humanized monoclonal antibody that selectively binds the negative regulatory region of the Notch1 receptor leading to inhibition of Notch1 signaling [15]. A phase I study has been conducted in subjects with solid tumors showing efficacy in cases with Notch1 pathway activation [16] . In this study, we investigated the role of the Notch ligands in activating Notch1 signaling in *NOTCH1*-mutated and - unmutated MCL cell lines and evaluated the effects of OMP-52M51 in these cell lines.

## Methods

### Cell lines and primary MCL cells

MCL cell lines Mino (CRL-3000), JeKo-1 (CRL-3006) and REC-1 (CRL-3004), were obtained from American Type Culture Collection (ATCC). To avoid Mycoplasma contamination, cell lines were routinely tested for Mycoplasma infection by PCR. The identity of all cell lines was verified by using GenePrint® kit (Promega, Madison, WI, USA). MCL cell lines were cultured in RPMI 1640 complemented with 10–20% fetal bovine serum (FBS), 2 mM L-glutamine and 50 μg/mL penicillin/streptomycin (Life Technologies, Carlsbad, CA, USA) and grown in a humidified atmosphere at 37 °C with 5% CO_2_. The murine bone-marrow derived stromal cell line OP9 (CRL-2749; ATCC) overexpressing DLL4 (OP9-DLL4) was generated and grown as described [17, 18]. Primary cells from MCL patients were isolated and cultured as described [19] and conserved within the Hematopathology collection of our institution registered at the Biobank from Hospital Clínic-IDIBAPS (R121004–094). The ethical approval for this project including informed patient consent was granted following guidelines of the Hospital Clínic Ethics Committee.

### Ligand stimulation and cell treatment

Four micrograms per milliliter of the recombinant Notch-receptor-ligands DLL1, DLL4, JAG1 and JAG2 (R&D Systems, Minneapolis, MN, USA) were resuspended in phosphate buffered saline (PBS) and stored in culture plates for 4 h at 4 °C to let them attach to the plates. MCL cells were treated with 25 μg/mL of OMP-52M51 or human IgG2 (both kindly provided by Oncomed Pharmaceuticals, Redwood City, CA, USA) and incubated at 37 °C for 2 h prior to adding them to the ligand-coated plates for stimulation. For coculture experiments, OP9 cells were plated overnight, and then medium was replaced by MCL cells (0.5 × 10^6^ cells/mL) previously treated for 2 h with OMP-52M51. After 24 h of coculture, MCL cells were collected by carefully rinsing the wells without disturbing the stroma monolayer and processed as required.

### Protein analysis

Whole-cell protein extracts were obtained using Triton containing lysis buffer (Tris-HCl pH 7.6 20 mmol/L, NaCl 150 mmol/L, EDTA 1 mmol/L, 1% Triton X-100) supplemented with protease and phosphatase inhibitors (10 μg/mL leupeptin, 10 μg/mL aprotinin, 1 mmol/L phenylmethanesulfonyl fluoride, 5 mmol/L NaF, 2 mmol/L Na_3_VO_4_). Solubilized proteins were quantified by Bradford protein assay and 50 μg of protein was analyzed by Western Blotting. The following primary antibodies were used: cleaved-NOTCH1 (Val1744)(D3B8), MEK1/2, phospho-MEK1/2 (Ser217/221)(41G9) (Cell Signaling, Boston, MA, USA), ERK1 (K-23), phospho-ERK (Thy202/204) (E-4) (Santa Cruz, Dallas, TX, USA), α-tubulin and β-actin (Sigma Aldrich, Saint Louis, MI, USA). Chemiluminescence was detected with ECL substrate (Pierce Biotechnology Waltham, MA, USA) on a mini-LAS4000 Fujifilm device (Fujifilm, Valhalla, NY, USA). Protein expression was densitometrically quantified using the Image Gauge software (Fujifilm).

### Immunohistochemistry and confocal microscopy

Lymph node (LN) biopsies from MCL cases were obtained from the Hematopathology collection of our institution registered at the Biobank from Hospital Clínic-IDIBAPS (R121004–094). Formalin-fixed paraffin-embedded (FFPE) tissue slides (serial 8 μm sections) were dewaxed and tissue antigens were retrieved by boiling for 10–15 min in sodium citrate (10 mM, pH 6.0). Slides were allowed to cool down to room temperature (RT) and then washed in distilled water and PBS. Quenching was carried out using 1% H_2_O_2_ in 100% methanol (40 min, RT) and permeabilization with 0,3% Triton-X-100 (20 min, Sigma-Aldrich) in PBS. Sections were incubated overnight with anti-human DLL4 (H-70) (Santa Cruz) and anti-human CD68 (Dako, Glostrup, Germany). Background staining was determined by incubating with irrelevant antibodies. Unspecific fluorescence was quenched by incubating with avidin/biotin blocking solutions (Vector Lab, Burlingame, CA, USA). For DLL4 detection, tissue slides were incubated with anti-rabbit IgG-HRP antibody (Dako). Signal was amplified using the Cyanine-3 Tyramide Signal Amplification Kit (TSA; NEL 744, Perkin Elmer, Waltham, MA). For CD68 detection, a biotinylated anti-mouse IgG (Vector Lab) was added following incubation with avidin/biotin complex (Elite Vectastain ABC Complex kit, Vector Lab). Signal was developed by adding Alexa-488-conjugated streptavidin. Nuclei were stained with Topro-3 (Invitrogen, Carlsbad, CA, USA) and mounted with Fluoromount-G (Southern Biotech., Birmingham, AL, USA). Images were acquired using a LSM510 laser scan confocal microscope (Zeiss, Oberkochen, Germany) coupled to an Axiovert200 (Zeiss) microscope, using 63x Plan-Neofluar magnification.

### Cell cycle assay

A total of 3 × 10^5^ MCL cells were collected after stimulation with DLL4 and incubated with OMP-52M51 for 48 h. Then cells were fixed in ice-cold 70% ethanol while being gently vortexed, incubated at − 20 °C for 24 h, washed in PBS and resuspended in 500 μL of staining solution containing 20 μg/mL propidium iodide (Invitrogen) and 100 μg/mL RNase A (Thermofisher) in PBS. Cells were incubated during 30 min at 37 °C and analysed using BD LSRFortessa 4 L cytometer (Becton Dickinson, Franklin Lakes, NJ, USA). Cell cycle analysis was performed using FlowJo software (Becton Dickinson).

### Chemotaxis assay

MCL cells were stimulated with DLL4 and incubated with OMP-52M51 for 48 h. Transwell culture polycarbonate inserts (6.5 mm diameter, 8 μm of pore size) (Corning, NY, USA) were transferred to wells containing 600 μL of RPMI supplemented with 0.5% BSA with 200 ng/mL of human recombinant CXCL12 (Peprotech). One hundred microliter of cell suspension (0.5 x 10^6^cells) was then added into the transwell inserts. Input cell count was obtained from adding 100 μL of cell suspension to wells containing 600 μL of 0.5% BSA in RPMI 1640. After 3 h, 100 μL were collected in triplicates from each lower chamber and input well, viable cells were counted on an Attune cytometer (Thermofisher, Waltham, MA, USA) under constant flow rate. Migration is represented as the ratio between migrated cells and total viable input cells.

### Human umbilical vein endothelial cells (HUVEC) tube formation assay

HUVEC, kindly provided by Dr. MC Cid (IDIBAPS), were cultured as described [20]. Supernatants from MCL cells (1 × 10^6^ cells/mL) were collected after 48 h of OMP-52M51 treatment of DLL4-stimulated cells. 24-well plates were coated with 300 μL of Matrigel (Becton Dickinson) before 500 μL of HUVEC (0.5 × 10^5^ cells/mL] and 500 μL of the supernatants were added. After 24 h, number of branch points was quantified as a measure of in vitro angiogenesis as the mean of five randomly chosen fields from each well. Images were taken with a DM IL LED microscope coupled to a DFC295 camera (magnification 100x) with Leica Application Suite v 3.7 software (Leica, Wetzlar, Germany).

### Gene expression profiling

Total RNA was extracted using the TRIzol method (Life technologies) according to manufacturer’s instructions. RNA integrity was examined with the Agilent 2100 Bioanalyzer (Agilent Technologies, Santa Clara, CA, USA) and only high quality RNA samples were further processed. RNA from MCL cell lines was hybridized to a Gene Chip HT HG-U219 array, according to Affymetrix (Santa Clara, CA, USA) standard protocols. RNA from MCL lymph nodes was hybridized to a Gene Chip Human Genome U133 plus 2.0 arrays, according to Affymetrix standard protocols. Determination of the detection call for each probe set of the array was obtained with GeneChip® Command Console® Software (AGCC) (Affymetrix). Raw data was normalized using the Robust Multichip Analysis (RMA) algorithm of the BioConductor Affy Package. Differential expression data analysis was carried out using the Multiexperiment Viewer Platform (TM4-MEV) [21]. The number of statistically significant up- and down-regulated genes was determined using Rank Products methodology [22] for cell lines, and Volcano plot [23] for MCL lymph nodes, both setting up a false discovery rate (FDR) < 0.2 and an absolute fold change (FC) > 1.75. PANTHER (http://pantherdb.org/) was used to perform gene ontology (GO) pathways analysis [24] to visualize the relationships between the significantly modulated genes of *NOTCH*-mutated MCL lymph nodes and proteins in known pathways of biological processes [25]. Primary microarray data of MCL cell lines and primary MCL lymph nodes are available at the Gene Expression Omnibus (GEO) of the National Center for Biotechnology Information under accession Nos. GSE125349, GSE36000 and GSE46969.

### Gene set enrichment analysis (GSEA)

For GSEA, the desktop application version 2.0 (GSEA, Broad Institute at MIT, Cambridge, MA; http://www.broadinstitute.org/gsea/) was applied using experimentally derived custom genes. The “Custom MCL” gene set was designed by manually grouping gene sets involved in pathways of Notch-activated genes in MCL according to the results described by Ryan et al. [26]. Briefly, we searched molecular signatures (http://software.broadinstitute.org/gsea/msigdb/) corresponding to the pathways described in the GO canonical pathways analysis and GSEA analysis of transcripts increased by Notch in Mino cells (except *MYC*), and the significant genes were grouped into specific pathways (Additional file 1: Table S1). The “NOTCH1 direct targets” gene set was also constructed based on the 79 genes described by Ryan et al. [26]. Similarly, the “NOTCH1 custom” gene set was designed by manually selecting significant Notch1 related genes found in the literature [6, 11, 15, 27–30]. Two-class analysis with 1000 permutations of gene sets and a weighted metric was used for all cases. The resulting gene sets with a false discovery rate (FDR) < 0.05 were considered to be significant, except for the “NOTCH1” gene set, where a FDR < 0.12 was used. GSEA analysis was performed of MCL cell lines stimulated or not with DLL4 and treated or not with OMP-52M51 and of MCL lymph node tissues with or without mutations in a *NOTCH* gene. HeatMaps were created using the Morpheus software (https://software.broadinstitute.org/morpheus/) followed by hierarchical clustering using one minus Pearson correlation of the average of gene expression in order to illustrate the differential expression of those genes significantly modulated by DLL4 stimulation and OMP-52M51 treatment in the MCL cell lines and by *NOTCH* gene mutation in MCL lymph node tissues for the all custom gene sets analysis performed.

### In vivo mouse model

NSG (NOD-scid-gamma) mice were injected intravenously (i.v.) with 10 × 10^6^ Mino cells. MCL cell engraftment was periodically monitored over a 3 months period. After 3 months, mice presented infiltration in several organs. Tumor cells from lymph nodes were collected, cultured in RPMI 1640 + 10% FBS as described above and cryopreserved after several passages. We next confirmed that these Mino cells engraft faster in a secondary transplant (45–60 days). Again, these cells obtained from lymph nodes were collected and cryopreserved. These “fast engrafting” tumor cells were then thawed and expanded to get enough cells for in vivo studies. 225 × 10^6^ Mino cells were then stimulated ex vivo by coculturing them with OP9-DLL4 cells (7.5 × 10^6^ Mino cells/plate 100 × 20 mm^2^ (Corning). After 24 h of incubation, 15 × 10^6^ stimulated Mino cells were injected into the intraperitoneal cavity (IC) of 12 female NSG mice at the age of 10 weeks. Mice were treated intraperitoneally 1 day prior to injection of cells and then every 4 days with 20 mg/kg of OMP-52M51 or control antibody human IgG2 (6 mice/group). After 10 days, mice were sacrificed and a peritoneal lavage (PL) was done by injecting the cavity with 5 mL of cold PBS. Human B-cells were purified by using human CD19 beads. Protein extracts were obtained and expression of cleaved Notch1 was analyzed by Western Blot. Procedures involving animals and their care are conforming to institutional guidelines that comply with national and international laws and policies (EEC Council Directive 86/609, OJ L 358, 12 December, 1987) and were authorized by the local ethical committee.

### Statistical analysis

Data is represented as the mean ± SD of 3 independent experiments. All statistical analyses were done by using GraphPad Prism 6.01 software (GraphPad Software, La Jolla, CA, USA). Volcano plot of *P* values as a function of weighted FC for mRNA was performed by using Multiplot Studio v1.5.20 software (Benooist-Mathis Lab, Harvard Medical School, MA, USA). Comparisons between 2 groups of samples were analyzed with Kruskall-Wallis nonparametric test followed by Mann-Whitney *U* test. Results were considered statistically significant when *P* < 0.05 (*P < 0.05; **P < 0.01; ***P < 0.001).

## Results

### Activation of Notch1 signaling can effectively be achieved by stimulation with DLL4 in *NOTCH1*-mutated MCL cells

A *NOTCH1*-mutation affecting the PEST-domain in exon 34 (p.Q2487*) was described in the established MCL cell line Mino [5, 6]. The effect of this mutation is considered to be ligand-dependent. In contrast, the MCL cell line REC-1 presented an intragenic deletion in exon 28 encoding for a truncated Notch1 protein with increased activity in a ligand-independent fashion [26, 31].

**Fig. 1:**
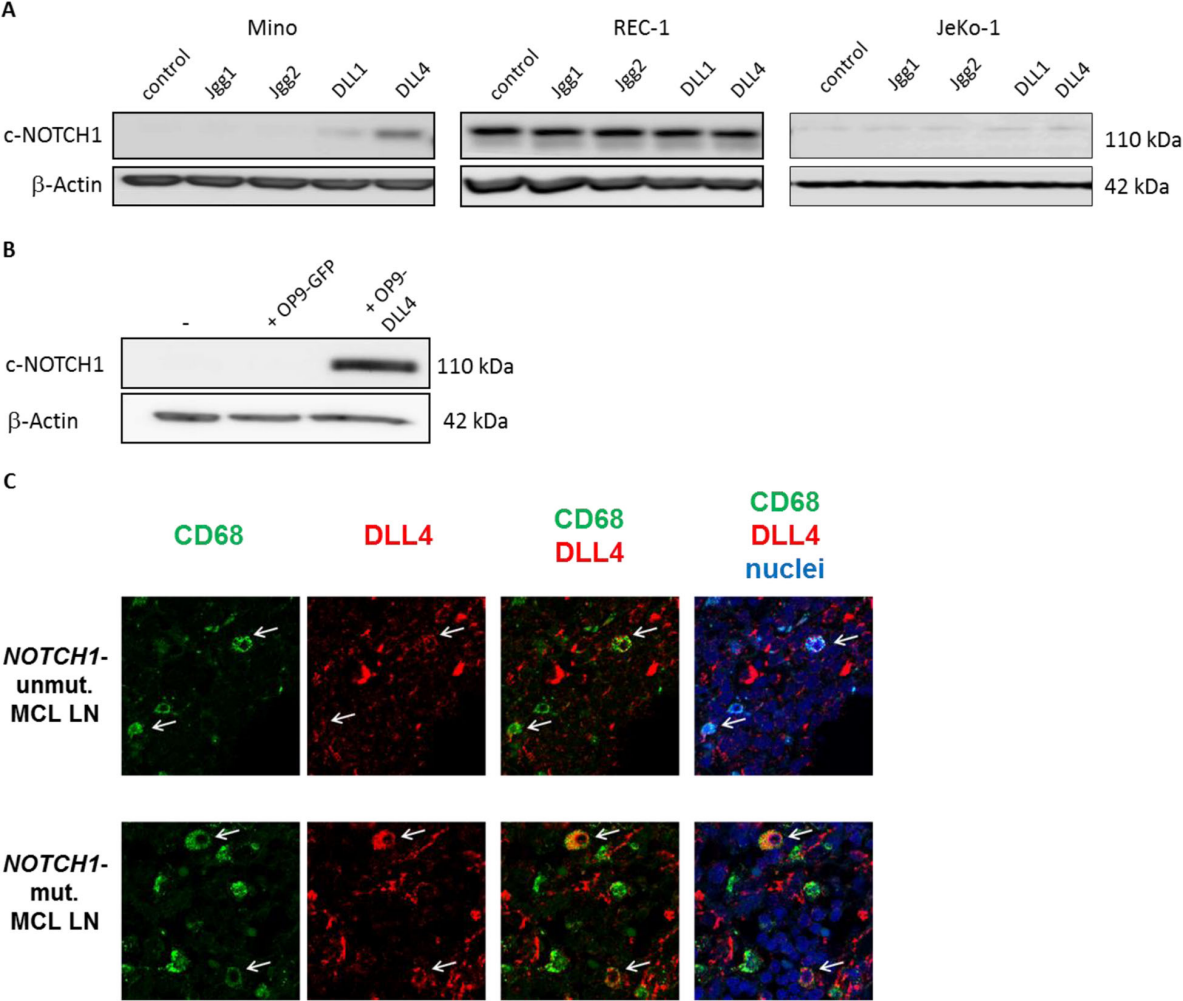
Activation of Notch1 signaling can effectively be achieved by stimulation with DLL4 in *NOTCH1*-mutated MCL cells. **a** Mino, REC-1 and JeKo-1 cells were stimulated with the different soluble ligands JAG1, JAG2, DLL1 and DLL4 (10 μg/mL). Protein expression levels of cleaved Notch1 were determined by Western Blot analysis after 48 h (*n* = 3, one representative experiment is shown). **b** Mino cells were cocultured with mesenchymal stem cells expressing DLL4 (OP9-DLL4) or GFP-transfected control cells for 48 h and protein expression of cleaved Notch1 was analyzed by Western Blot. [(*n*=3, one representative experiment is shown). **c** Double staining of MCL lymph node (LN) with anti-CD68 (marker of histiocytes, green) and anti-DLL4 (red). Nuclei were stained with Topro-3 (blue). Images were acquired using a LSM510 laser scan confocal microscope coupled to an Axiovert 200 microscope, using 63x Plan-Neofluar magnification. Images from a representative case per group are shown

First, we analyzed which Notch ligand was the most effective to stimulate Notch1 signaling in MCL. *NOTCH1*-mutated cell lines Mino and REC-1 and the *NOTCH1*-wt cell line JeKo-1 were stimulated with the recombinant ligands DLL1, DLL4, JAG1 and JAG2 and the Notch activation status in these samples was determined by Western Blot analysis of cleaved Notch1. As shown in Fig. 1a, DLL4 and DLL1 activated the expression of cleaved Notch1 in Mino cells, DLL4 being the most potent ligand. In contrast, these ligands induced only a minor Notch1 activation in unmutated JeKo-1 cells. We confirmed that REC-1 cells overexpressed cleaved Notch1 independently of the ligands. The effect of DLL4 was then confirmed by coculturing of Mino cells with the bone-marrow derived mesenchymal stem cell line OP9 overexpressing DLL4 (OP9-DLL4), which represents a cell culture model better reflecting the situation in vivo, where Notch1 ligands are usually presented by microenvironmental cells [26, 31]. Increased expression of cleaved Notch1 in Mino cells upon coculture with OP9-DLL4 cells was detected by Western Blot (Fig. 1b). In view of the remarkable in vitro effect of DLL4 in *NOTCH1*-mutated MCL, we sought to characterize which cells could express this ligand and trigger Notch activation in vivo. We therefore analyzed the expression of DLL4 in MCL lymph nodes (LN) using immunofluorescence staining and confocal microscopy. We found that DLL4 was widely expressed in the vascular endothelium (data not shown) and, importantly, it was expressed by some histiocytic (CD68+) cells in both *NOTCH1*-mutated and -unmutated cases (Fig. 1c).

### Treatment of *NOTCH1*-mutated MCL cells with OMP-52M51 effectively prevents DLL4-dependent activation of Notch1

We next investigated the effect of the humanized monoclonal Notch1 antibody OMP-52M51 in *NOTCH1*-mutated (Mino) and unmutated (JeKo-1) MCL cell lines. Since the recognition-binding site of OMP-52M51 in the EGF-like domain is missing in the REC-1 cell line due a deletion in this site [26], this cell line was not suitable for further investigation.

**Fig. 2:**
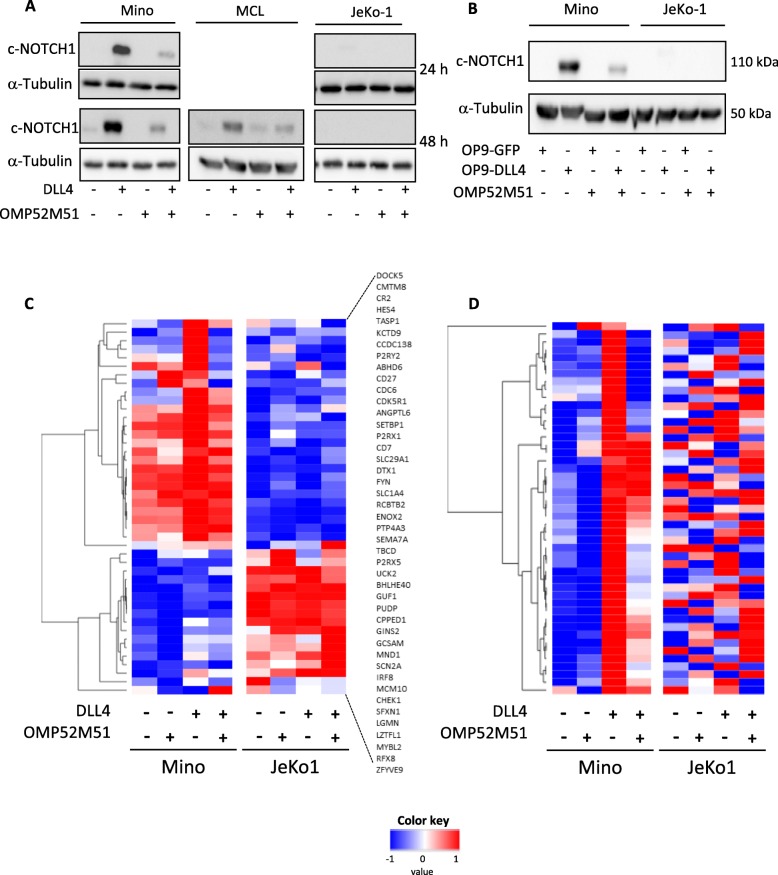
Treatment of *NOTCH1*-mutated MCL cells with OMP-52M51 effectively prevents DLL4-dependent activation of Notch1. **a** Mino and JeKo-1 MCL cells and *NOTCH1*-mutated primary MCL cells (patient sample MCL) were treated with OMP-52M51 or IgG2 control antibody and stimulated with human recombinant DLL4 (4 μg/mL). Protein expression of cleaved Notch1 was determined by Western Blot analysis after 24 and 48 h. **b** Mino and JeKo-1 MCL cells were treated with OMP-52M51 and cocultured with DLL4-overexpressing OP9-stromal cells or GFP-transfected control cells. After 48 h, Western Blot analysis of cleaved Notch1 protein expression was performed. **c** Significantly modulated genes (FDR < 0.123; *p* < 0.005; NES > 1.48) related to the Notch1 signaling pathway (NOTCH custom) upon stimulation of Mino and JeKo-1 cells with DLL4 after 48 h obtained with the Gene Set Enrichment Analysis (GSEA) software. **d** Significantly upregulated genes (FDR < 0.001; *p* < 0.001, NES = 3.00) upon stimulation of Mino and JeKo-1 cells with DLL4 after 48 h obtained with GSEA applying a signature of Notch target genes described in MCL cells (NOTCH direct targets). Heatmaps were hierarchical clustered by one minus Pearson correlation of the average of gene expression

We observed that treatment of DLL4-stimulated Mino cells with OMP-52M51 for 24 and 48 h resulted in inhibition of DLL4-mediated cleaved-Notch1 overexpression. This effect could also be observed in primary cells from a *NOTCH1*-mutated MCL case [MCL#1] carrying the typical 2-bp deletion in exon 34 (c.7541_7542delCT) (Fig. 2a). We confirmed the potential of OMP-52M51 to inhibit DLL-stimulated induction of Notch1 signaling also in the coculture system of Mino cells with OP9-DLL4 (Fig. 2b). Again, no effect was detected in the *NOTCH1*-wt JeKo-1 cell line.

To evaluate the effect of OMP-52M51 on downstream signaling of Notch1, we performed a gene expression profile analysis in JeKo-1 and Mino cells stimulated with DLL4 and treated with OMP-52M51 for 48 h. Using the GSEA software analyzing a customized *NOTCH1* set of genes (*NOTCH1* custom) [6, 11, 15, 27–30], we detected 44 significantly upregulated leading edge genes (Fig. 2c, Additional file 2: Table S2) upon DLL4-treatment in Mino cells (FDR < 0,123; *p* < 0,005, NES > 1.48). As displayed in the Z score heatmap, the significantly modulated genes upon DLL4 stimulation in Mino cells were effectively downregulated by treatment with OMP-52M51 (Fig. 2c).

When we applied a signature of *NOTCH1* target genes described in MCL cells (*NOTCH1* direct targets) [26], a strong upregulation of these genes was detected in Mino cells stimulated with DLL4 (FDR < 0.001; *p* < 0.001, NES = 3.00). A good correlation was also observed in JeKo-1 stimulated with DLL4 (FDR = 0.025; *p* = 0.025, NES = 1.40) (Additional file 3: Table S3 and Figure S1), confirming again that in *NOTCH1*-unmutated cells the activation of Notch signaling is less potent than in mutated cells. This may be due to the fact that the signaling would not be sustained enough as the wild type Notch1 protein has a rapid turnover [7]. Again, OMP-52M51 was able to revert the expression of these direct Notch-target genes in Mino cells (Fig. 2d).

### OMP-52M51 significantly impedes DLL4-induced upregulation of genes involved in lymphoid biology, lymphomagenesis and disease progression

**Fig. 3:**
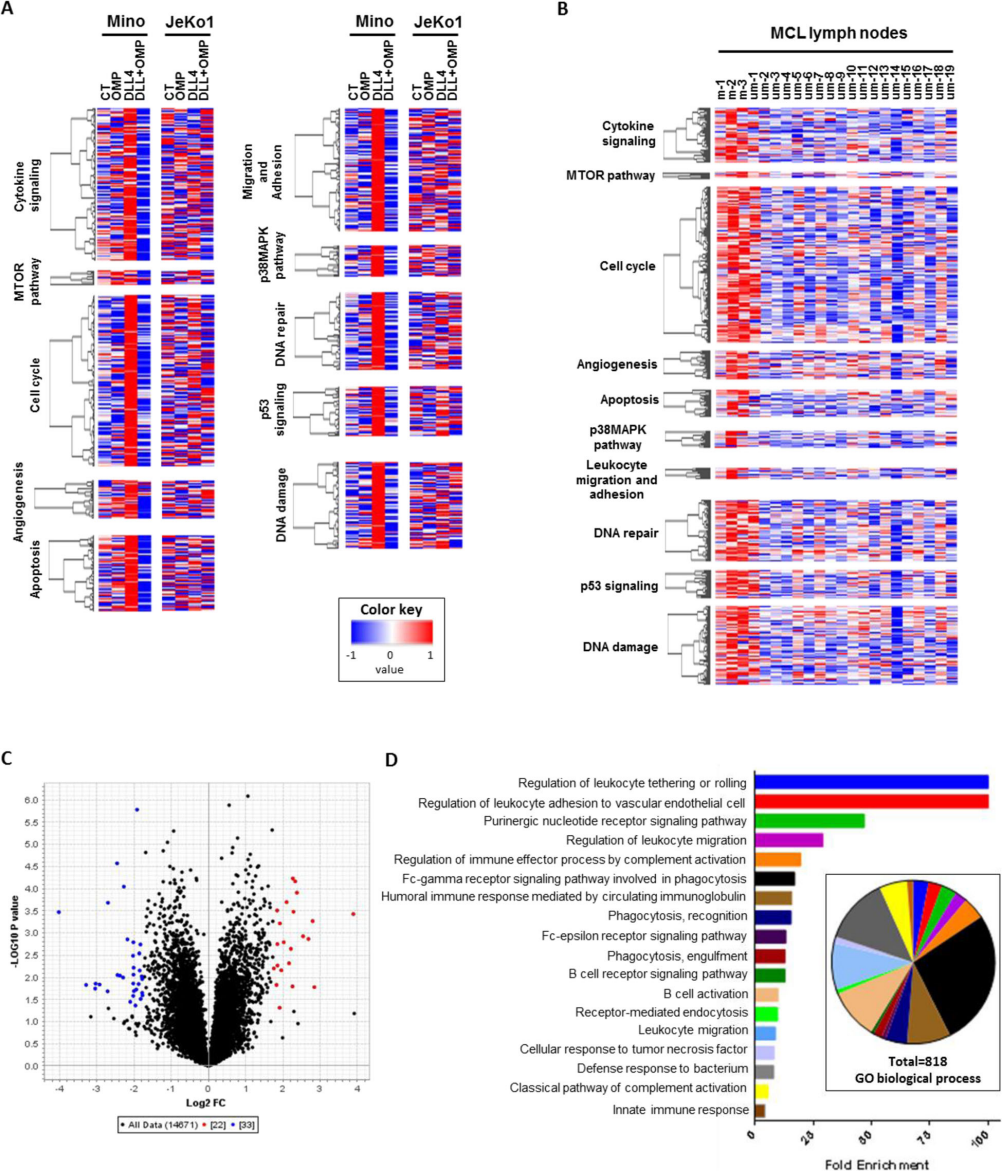
OMP-52M51 significantly impedes DLL4-induced upregulation of numerous genes involved in lymphoid biology, lymphomagenesis and disease progression mimicking the signature of *NOTCH* mutated MCL lymph nodes. Heatmaps displaying the significantly upregulated genes and grouped by pathway signaling obtained after GSEA analysis (Custom MCL set of genes) of (**a**) Mino cells treated with OMP-52M51 after DLL4-stimulation and (**b**) MCL lymph nodes with or without mutation in *NOTCH* genes. **c** Volcano plot filtering of 55 genes differentially expressed in *NOTCH*-mutated MCL lymph nodes. **d** Enrichment of biological processes found by PANTHER gene ontology analysis of the volcano-filtered genes differentially expressed in *NOTCH*-mutated MCL lymph nodes

Our gene expression results were analyzed by GSEA using a custom gene set (Custom MCL) generated with data obtained from an integrative analysis of Notch-regulated transcripts, genomic binding of Notch transcription complexes and genome conformation data in MCL cell lines [26]. We observed that DLL4 upregulated genes related to angiogenesis, apoptosis, migration and adhesion, cell cycle, cytokine signaling, DNA damage and repair, MTOR and MAPK signaling, leukocyte proliferation and defense response (Fig. 3a). OMP-52M51 was able to modulate all these gene sets only in the *NOTCH1*-mutated Mino cell line (Additional file 4: Table S4).

Next, we analyzed the gene expression profile of lymph nodes from primary MCL cases (*n* = 21), 3 of them harboring *NOTCH1/2* mutations. By using the same GSEA analysis, in lymph nodes from primary MCL cases with *NOTCH1/2* mutations, we detected an upregulation of the same gene sets as observed in DLL4-stimulated Mino cells (Fig. 3b, Additional file 5: Table S5). In these cases, to maintain Notch signaling, a crosstalk between tumor MCL cells and accessory cells, probably histiocytes, is required. In addition, the PANTHER GO analysis of the 55 genes differentially expressed in MCL lymph nodes carrying *NOTCH* gene mutations compared to *NOTCH*-unmutated MCL lymph nodes, filtered by Volcano plot (Fig. 3c, Additional file 6: Table S6), revealed 818 signaling pathways associated with leukocyte biology primarily enriched for biological processes associated with disease progression such as regulation of tethering/rolling, adhesion and migration of leukocytes (Fig. 3d).

**Fig. 4:**
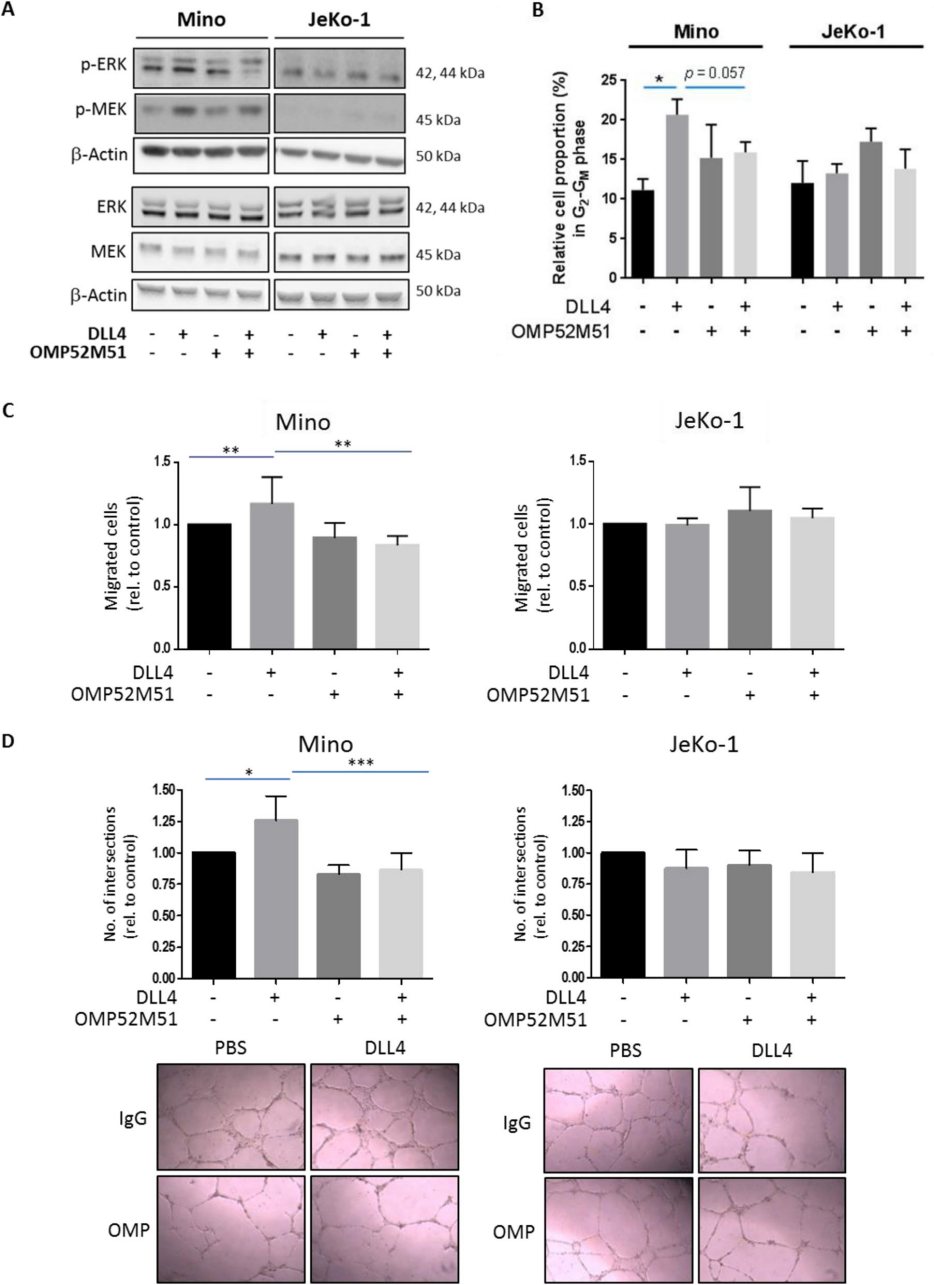
Activating *NOTCH1* mutations promote BCR signaling, proliferation, angiogenesis and enhance tumor cell migration upon DLL4-stimulation that can be abolished by treatment with OMP-52M51. **a** Effect of DLL4-stimulation and treatment with OMP-52M51 on ERK and MEK phosphorylation. Mino and JeKo-1 MCL cells were stimulated with DLL4 (4 μg/mL) and treated with OMP-52M51 or IgG2. After 48 h, protein expression of p-ERK, total ERK1, p-MEK1/2 and total MEK1/2 was assessed by Western Blot [*n* = 3; one representative experiment is shown]. **b** Relative cell proportion in G_2_-G_M_ phase of cell cycle in Mino and JeKo-1 cells stimulated or not with DLL4 and treated with OMP-52M51 or IgG2 for 48 h. Cell cycle phases were measured by flow cytometry using propidium iodide and analysed using FlowJo software (*n* = 4, *p = 0.0286, bars represent the mean ± SD). **c** Mino and JeKo-1 cells were stimulated with DLL4 and treated with OMP-52M51 or IgG2 for 48 h. Migration of cells was assessed in a transwell system with inserts of 8 μm pore size. Migration was defined by counting the cells that migrated to the lower chambers containing medium with the chemoattractant CXCL12 (200 ng/mL) (*n* = 5, **p = 0.0079, bars represent the mean ± SD). **d** Cells were stimulated with DLL4 and treated with OMP-52M51 or IgG2 for 48 h. Supernatants were then harvested and added to HUVEC. After 24 h, the number of branch points was quantified as the mean of five randomly chosen fields from each well. Pictures were taken with a DM IL LED microscope coupled to a DFC295 camera (magnification 100x) (*n *= 5, bars represent the mean ± SD, *p = 0.05; ***p = 0.001). Microscope images from one representative experiment are shown

Given that most of these gene sets were related to B cell activation, regulation of leukocyte tethering or rolling and leukocyte adhesion and migration, we selected these signatures for further functional validation. As the activation of ERK is an important integration point for B cell activation [32], we analyzed phosphorylation of ERK1/2 and MEK by Western Blot after stimulation of Mino and JeKo-1 cells with DLL4 and treatment with OMP-52M51. As displayed in Fig. 4a, DLL4 stimulation increased phosphorylation of both, MEK and ERK in Mino cells, indicating that aberrant Notch1 signaling stimulates B cell activation in MCL. This effect could be reduced by treatment with OMP-52M51, a process that was not observed in *NOTCH1*-unmutated JeKo-1 cells (Fig. 4a). To corroborate whether the upregulation of genes related with cell cycle is effectively dependent of aberrant expression of *NOTCH1* genes, we analyzed the modulation of cell cycle in *NOTCH1*-mutated Mino cells and *NOTCH1*-unmutated JeKo-1 cells treated or not with DLL4 and/or OMP-52M51. We observed that DLL4-stimulated Mino cells showed a significant increase of cell proportion in G_2_-G_M_ phase that was partially reverted (*p* = 0.057) by incubation with OMP-52M51 for 48 h (Fig. 4b).

We next studied the effect of aberrant Notch1 signaling on cell migration by means of chemotaxis assays. As shown in Fig. 4c, DLL4 stimulation of Notch1 significantly increased CXCL12-induced migratory capacity of Mino cells that could be abrogated by treatment with OMP-52M51. In contrast, no effect could be observed in unmutated JeKo-1 cells (Fig. 4c).

The DLL4-Notch1 signaling pathway plays an important role in regulating blood vessel formation during physiological and pathological angiogenesis [33, 34]. Furthermore, data from our gene expression arrays revealed that DLL4 stimulation induces several genes related to angiogenesis. This prompted us to investigate the impact of activating *NOTCH1* mutations on tumor angiogenesis. We therefore used supernatants of Mino and JeKo-1 cells stimulated or not with DLL4 and treated or not with OMP-52M51 in a tube formation assay with HUVEC. Supernatants of DLL4 stimulated *NOTCH1*-mutated Mino cells significantly increased number of branch points as a measure of in vitro angiogenesis compared to those of unstimulated cells. Importantly, the proangiogenic effect of these supernatants could be effectively abolished by treatment of cells with OMP-52M51. Again, no differences were detected in unmutated JeKo-1 cells (Fig. 4d).

### OMP-52 M51 effectively inhibits DLL4 induced activation of Notch1 in an in vivo model

**Fig. 5:**
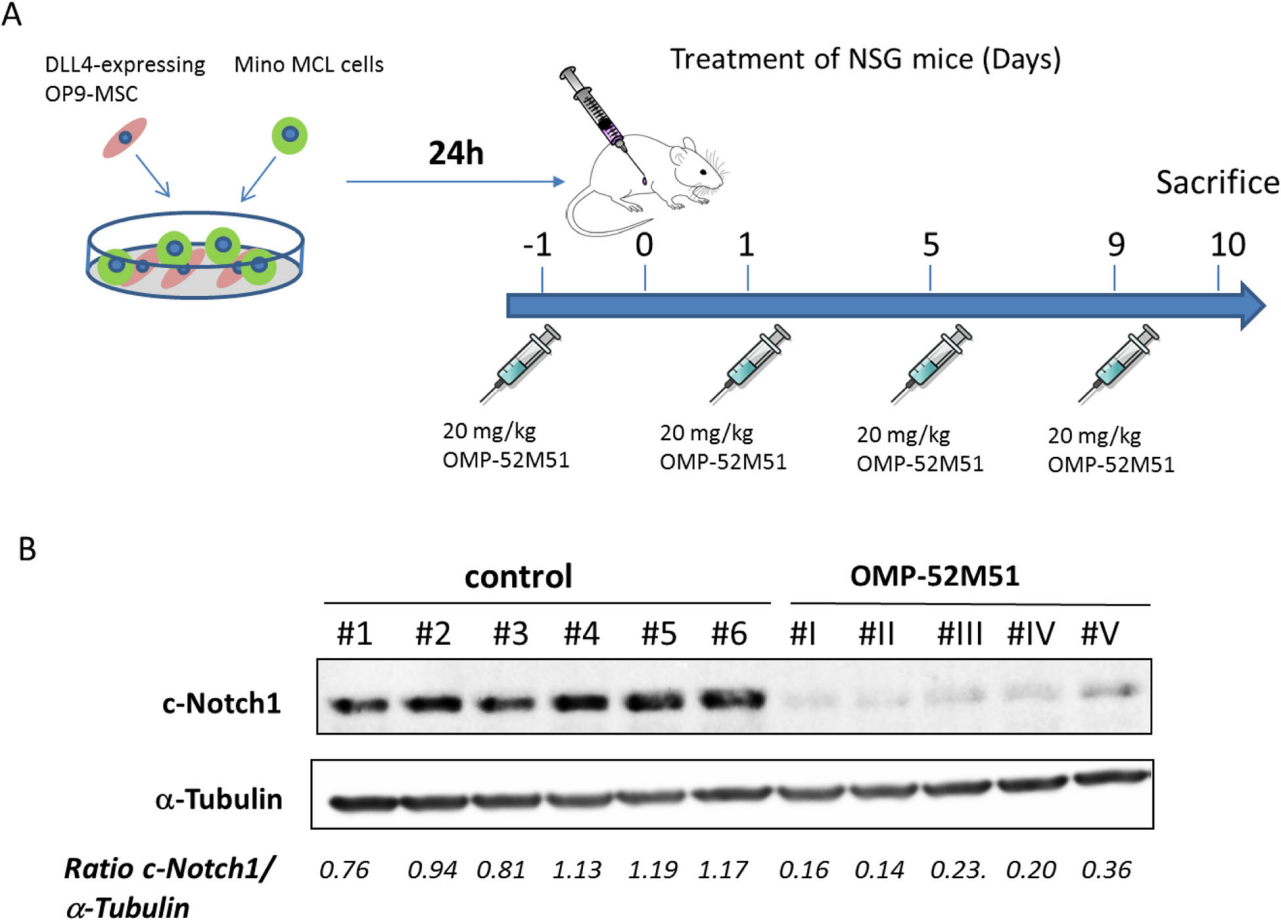
OMP-52M51 effectively inhibits DLL4 induced activation of Notch1 in an in vivo model. **a** Mino cells were stimulated ex vivo by coculturing them with DLL4 expressing stromal cells. After stimulation for 24 h, cells were injected into the intraperitoneal cavity of NSG mice. Mice were treated IP 1 day prior to injection of cells and then every 4 days with 20 mg/kg of OMP-52M51. After 10 days, mice were sacrificed and cells were recollected from the intraperitoneal cavity. **b** Expression of cleaved Notch1 was analyzed by Western Blot

Finally, we aimed to confirm the activity of OMP-52M51 in an in vivo MCL model. As *NOTCH1*-mutated Mino cells are dependent on ligand activation, these cells were stimulated ex vivo with OP9-DLL4 cells to ensure human Notch1 activation. After stimulation of Mino cells for 24 h, cells were injected into the IC of NSG mice and treated every 4 days with 20 mg/kg of OMP-52M51. After 10 days, mice were sacrificed and cells were recollected from the IC (Fig. 5a). Total recovery of the peritoneal cells was evaluated by flow cytometry after staining with huCD45/CD19/antibodies. This short-term treatment with the anti-Notch1 antibody did not affect cell viability or tumor cell counts (data not shown). Protein extracts were obtained and Western Blot analysis confirmed that OMP-52M51 was able to inhibit cleaved Notch1 in vivo (Fig. 5b), although this short-term OMP-52M51 treatment was not enough to cause a significant efficacy in tumor growth.

## Discussion

Activating *NOTCH*1 mutations are among the most frequent secondary genetic alterations in MCL detected in 5–10% of cases [5, 6, 35]. Similar to CLL, the majority of *NOTCH1* alterations described in MCL are nonsense truncating mutations and small frame-shift indels located in exon 34 which encodes the PEST domain, resulting in enhanced Notch1 signaling [7, 30]. The clinical significance of this mutation is highlighted by the fact that patients harboring a *NOTCH1*-mutation had significantly shorter overall survival rates [5, 6]. Yet, the molecular impact of *NOTCH1* mutations in MCL is not well understood. Recently, it has been postulated that Notch signaling regulates genes involved in BCR and cytokine signaling as well as the oncogene *MYC*, which sustains proliferation of Notch-dependent MCL cell lines [26]. Targeting Notch signaling has been studied in various cancer types [6, 10, 36], and particularly in hematological malignancies carrying *NOTCH1* mutations [10, 37]. It has been reported that GSI-mediated inhibition of Notch signaling in *NOTCH1*-mutated MCL cells resulted in reduction of cell proliferation and apoptosis induction [6]. However, the clinical applicability of GSI is limited due to severe gastrointestinal toxicities [12, 13]. Thus, alternative strategies for targeting Notch signaling are highly warranted. In this way, we analyzed the effect of a humanized IgG2 monoclonal antibody OMP-52M51 that showed encouraging antitumor efficacy in T-acute lymphoblastic leukemia (ALL) xenograft models [15]. Recently, phenotypic and molecular features of resistance after long-term treatment with OMP-52M51 have been reported, that are highly heterogeneous, suggesting that leukemia cells can adopt several strategies to evade Notch inhibition according to the therapeutic drug used [38].

In Non-Hodgkin lymphomas, only little is known about the dependency of Notch1 signaling activation upon a certain ligand. Candidate cellular sources include other hematopoietic cells; endothelial cells, which are capable of inducing Notch signaling in B lymphoma cells [39] and DLL1/4-expressing fibroblastic cells in secondary lymphoid organs [40]. In this study, we show for the first time that DLL4 is a potent ligand to activate Notch1 signaling in MCL cells harboring a *NOTCH1* mutation in the PEST domain, whereas JAG1 and JAG2 could not sufficiently stimulate Notch1 activity. DLL4 was shown to be constitutively expressed in some lymphoid organs, where it influences regulation of Notch signaling during hematopoiesis [41]. In T-ALL cells, where mutations in *NOTCH1* are frequent and well characterized, DLL4 plays an important role as part of the tumor microenvironment contributing to early steps of T-ALL cell growth [42]. Although immobilized DLL1 was also used to model ligand-dependent Notch1 activation in MCL [26], the effect of DLL1 in MCL cells is lower than of DLL4. Only a minor effect upon any ligand stimulation was observed in *NOTCH1-*wt cells corroborating the fact that *NOTCH1* mutations in the PEST domain lead to a more stabilized protein due to loss of the recognition site from the ubiquitin ligase degradation complex [7]. Furthermore, we provide evidence that DLL4 is expressed in the lymph node MCL compartment, where it could promote Notch activation. In particular, we detected DLL4 to be highly expressed in the vascular endothelium and by some CD68-positive cells, indicating that DLL4 expressed by the LN microenvironment might provide a specific niche for Notch activation.

Furthermore, our results confirm that stimulation of Notch signaling by Delta-like ligands could have a critical role in MCL pathogenesis as we observed upregulation of several direct Notch target genes [26] involved in angiogenesis, apoptosis, migration and adhesion, cell cycle, cytokine signaling, DNA damage and repair, MTOR and MAPK signaling, leukocyte proliferation and defense response of B cell activation, cell cycle progression and oncogenesis both in DLL4-stimulated Mino cells and in lymph nodes from primary MCL cases carrying *NOTCH* mutations. In this way, we detected the induction of transcription factors genes implicated in B-cell differentiation and activation, *PAX5* and *IRF8* [43–46] as well as of *MYBL2*, a transcription factor participating in cell cycle progression [47, 48] and recently described as a direct Notch1 target in B cell lymphomas [26]. We confirmed a possible interaction between Notch and BCR signaling in B cell malignancies [26] as we observed the upregulation of several genes related to B cell activation (*FYN, FGR, NEDD9* and *SH2B2*) and an increase of cell proliferation after DLL4 stimulation in *NOTCH1*-mutated MCL cells. Moreover, the results obtained for *NOTCH* -mutated MCL lymph nodes could be considered a proof-of-concept for those observed in DLL4-stimulated Mino cells, and are in concordance with the poor overall survival [6] and prognosis [5] associated with *NOTCH* mutations in MCL. Our next aim was to analyze the potential effect of the anti-Notch1 antibody OMP-52M51 on blocking the *Delta-like* ligand-induced signal in MCL. Treatment of Mino cells with OMP-52M51 could effectively inhibit DLL4-dependent Notch1-activation and suppress transcriptional expression of several direct Notch target genes described in MCL [26]. These findings are in line with results observed in T-ALL, where OMP-52M51 treatment of T-ALL patient samples harboring mutations in the *NOTCH1-*PEST-domain caused strong inhibitory effects on the expression of Notch-target genes [15]. Furthermore, we confirmed that in our model anti-Notch1 therapy attenuated the expression levels of the four well known Notch-target genes that have been described as a marker of the effect of OMP-52M51 in T-ALL (*CR2, DTX1, HES1* and *HES4*) [15]. Moreover, we elucidated a functional relationship between Notch1 signaling and microenvironment processes related to MCL aggressiveness such as cell proliferation, cell migration and angiogenesis. Notch1 signaling has been shown to play a role in CCL19-driven homing of CLL cells [49] and Notch1 signaling inhibition in multiple myeloma was described to prevent tumor cell migration [50]. Accordingly, we showed that OMP-52M51 reverts the strong induction of gene signatures related to tumor cell migration and adhesion upon Notch1 activation and prevents DLL4-stimulated migratory capacity of MCL cells.

The DLL4-Notch1 axis is known to play an important role in regulating angiogenesis. Previous studies have shown that productive tumor angiogenesis requires cooperation between VEGF-A, which induces proliferation of endothelial ‘tip’ cells and expression of DLL4 in ‘stalk’ cells [51]. In this context, DLL4 inhibits endothelial proliferation and promotes branching morphogenesis, and the balance between proliferation and branching is key to the formation of a functional capillary network. As such, treatment with anti-DLL4 antibodies resulted in disorganized angiogenesis, characterized by endothelial proliferation without formation of functional capillaries [52]. In line with this, we showed that supernatants of *NOTCH1*-mutated MCL cells stimulated with DLL4 increased HUVEC tube formation, whereas OMP-52M51 blocked this proangiogenic effect. We therefore postulate that by promoting pronounced vasculature development, DLL4-stimulated Notch1 signaling might contribute to the clinically observed aggressive behavior of *NOTCH1*-mutated MCL. Interestingly, *NRARP*, one of the genes with the strongest induction upon Notch1 stimulation in our gene expression array, has been described to be related to Notch signaling [53] and shown to act as a molecular link between Notch and Wnt signaling in endothelial cells to control stability of new vessel connections [54]. In this sense, our results suggest that in MCL, the link between Notch1 and NRARP might promote angiogenesis and needs to be further explored.

Importantly, we showed that even if DLL4 could potentially activate Notch1 signaling irrespective of the mutational status of *NOTCH1*, its functional effects are specific for *NOTCH1*-mutated MCL cells. This might be due to the fact that in *NOTCH1*-unmutated MCL, the weak expression of intracellular cleaved Notch1 upon ligand-activation seems to be very unstable due to rapid proteasomal degradation and might not be potent enough to cause functionally relevant transcriptional effects. This observation is of clinical relevance as a specific Notch1-antibody therapy might be a promising therapeutic alternative for the subgroup of patients with *NOTCH1*-PEST-mutations**.**

## Conclusion

We show for the first time that DLL4 is a potent stimulator of Notch1 signaling in *NOTCH1*-mutated MCL and that expression of this ligand observed in histiocytic cells from MCL lymph nodes might provide a specific niche for Notch activation. We propose a link between Notch1-induced expression of tumor-promoting genes and activation of processes contributing to a more aggressive MCL phenotype. Furthermore, our findings indicate that specific inhibition of the Notch1-ligand-receptor interaction provides an efficient and specific therapeutic alternative for a subset of MCL patients.

## Supplementary information

**Additional file 1: Table S1.** Gene sets included in the “Custom MCL” set of genes.

**Additional file 2: Table S2.** Significantly regulated *NOTCH1* target genes upon DLL4 stimulation using a customized *NOTCH1* set of genes (NOTCH1 custom) [6, 11, 15, 27–30]

**Additional file 3: Table S3.** Significantly regulated direct *NOTCH1* target genes upon DLL4 stimulation using a customized set of genes according to Ryan et al. (NOTCH1 direct targets) [26]. **Figure S1.** GSEA Enrichment plots upon DLL4 stimulation in Mino and JeKo-1 cells using a customized set of genes according to Ryan et al. (NOTCH1 direct targets) [26]

**Additional file 4: Table S4.** Modulated gene sets upon treatment with OMP-52M51 of DLL4-stimulated Mino cells using a customized set of genes (Custom MCL)

**Additional file 5: Table S5.** Modulated gene sets comparing *NOTCH*-mutated and wild type lymph nodes from MCL patients using a customized set of genes (Custom MCL)

**Additional file 6: Table S6.** Significantly modulated genes (FC=1.75) in lymph nodes from *NOTCH*-mutated MCL patients

## Data Availability

The datasets used and/or analyzed during the current study are available from the corresponding author on reasonable request. Primary microarray data are available at the Gene Expression Omnibus (GEO) of the National Center for Biotechnology Information Nos. GSE125349, GSE36000 and GSE46969.

## Abbreviations

ALL: Acute lymphoblastic leukemia
ATCC: American Type Culture Collection
BCR: B-cell receptor
Bhlh: Basic-helix-loop-helix
Ct: Cycle threshold
DLL: Delta like ligands
FBS: Fetal bovine serum
FC: Fold change
FDR: False discovery rate
FFPE: Formalin-fixed paraffin-embedded
GEO: Gene Expression Omnibus
GSEA: Gene Set Enrichment analysis
GSI: Gamma-secretase inhibitors
IC: Intraperitoneal cavity
IgG2: Immunoglobulin G2
JAG: Jagged ligands
LN: Lymph nodes
MCL: Mantle cell lymphoma
NICD: NOTCH1 Intracellular domain
NSG: NOD Scid Gamma
PBS: Phosphate buffered saline
RMA: Robust Multichip Analysis
RT: Room temperature
