# Supplementary material for: Notch1 signaling in NOTCH1-mutated mantle cell lymphoma depends on Delta-Like ligand 4 and is a potential target for specific antibody therapy
Source: J Exp Clin Cancer Res. 2019 Nov 1;38:446. doi: 10.1186/s13046-019-1458-7 (PMC6825347; doi:10.1186/s13046-019-1458-7)
Supplement: Supplementary file 1 — Additional file 1: Table S1. Gene sets included in the “Custom MCL” set of genes. [file 13046_2019_1458_MOESM1_ESM.pdf]

Additional file1: Table S1. Gene sets included in the “Custom MCL” set of genes

Custom gene set name	# genes grouped	Gene sets source	Description
Cytokine signaling	309	BIOCARTA_IL1R_PATHWAY	> Signal transduction through IL1R
		BIOCARTA_IL2_PATHWAY	> IL 2 signaling pathway
		BIOCARTA_IL6_PATHWAY	> IL 6 signaling pathway
		BIOCARTA_IL7_PATHWAY	> IL-7 Signal Transduction
		GO_CYTOKINE_RECEPTOR_ACTIVITY	> Combining with a cytokine and transmitting the signal from one side of the membrane to the other to initiate a change in cell activity.
		REACTOME_CYTOKINE_SIGNALING_IN_IMMUNE_SYSTEM	> Genes involved in Cytokine Signaling in Immune system
Cell Cycle	353	BIOCARTA_CELLCYCLE_PATHWAY	> Cyclins and Cell Cycle Regulation
		BIOCARTA_G1_PATHWAY	> Cell Cycle: G1/S Check Point
		BIOCARTA_P27_PATHWAY	> Regulation of p27 Phosphorylation during Cell Cycle Progression
		CELL_CYCLE_CHECKPOINT_GO_0000075	> Genes annotated by the GO term GO:0000075. A point in the eukaryotic cell cycle where progress through the cycle can be halted until conditions are suitable for the cell to proceed to the next stage.
		CELL_CYCLE_ARREST_GO_0007050	> Genes annotated by the GO term GO:0007050. Any process by which progression through the cell cycle is halted during one of the normal phases (G1, S, G2, M).
		CELL_CYCLE_GO_0007049	> Genes annotated by the GO term GO:0007049. The progression of biochemical and morphological phases and events that occur in a cell during successive cell replication or nuclear replication events. Canonically, the cell cycle comprises the replication and segregation of genetic material followed by the division of the cell, but in endocytes or syncytial cells nuclear replication or nuclear division may not be followed by cell division.
		CELL_CYCLE_PHASE	> Genes annotated by the GO term GO:0022403. A cell cycle process comprising the steps by which a cell progresses through one of the biochemical and morphological phases and events that occur during successive cell replication or nuclear replication events.
Angiogenesis	54	ANGIOGENESIS	> Genes annotated by the GO term GO:0001525. Blood vessel formation when new vessels emerge from the proliferation of pre-existing blood vessels.
		ROZANOV_MMP14_TARGETS_SUBSET	> Genes linked to the ECM maintenance and angiogenesis that were changed in HT1080 cells (fibrosarcoma) over-expressing MMP14 [GeneID=4323] compared to those with knockdown of the gene by RNAi.
		HU_ANGIOGENESIS_DN	> Down-regulated genes that separate angiogenic from non-angiogenic non-small cell lung carcinoma (NSCLC) samples.
		HU_ANGIOGENESIS_UP	> Up-regulated genes that separate angiogenic from non-angiogenic non-small cell lung carcinoma (NSCLC) samples.
Apoptosis	147	BIOCARTA_P53_PATHWAY	> p53 Signaling Pathway
		GO_EXTRINSIC_APOPTOTIC_SIGNALING_PATHWAY	> A series of molecular signals in which a signal is conveyed from the cell surface to trigger the apoptotic death of a cell. The pathway starts with either a ligand binding to a cell surface receptor, or a ligand being withdrawn from a cell surface receptor (e.g. in the case of signaling by dependence receptors), and ends when the execution phase of apoptosis is triggered.
		GO_INTRINSIC_APOPTOTIC_SIGNALING_PATHWAY	> A series of molecular signals in which an intracellular signal is conveyed to trigger the apoptotic death of a cell. The pathway starts with reception of an intracellular signal (e.g. DNA damage, endoplasmic reticulum stress, oxidative stress etc.), and ends when the execution phase of apoptosis is triggered. The intrinsic apoptotic signaling pathway is crucially regulated by permeabilization of the mitochondrial outer membrane (MOMP).
		REACTOME_APOPTOSIS	> Genes involved in Apoptosis
		SA_CASPASE_CASCADE	> Apoptosis is mediated by caspases, cysteine proteases arranged in a proteolytic cascade.
MTOR	17	BIOCARTA_MTOR_PATHWAY	> mTOR Signaling Pathway
		KEGG_MTOR_SIGNALING_PATHWAY	> mTOR signaling pathway
Migration and Adhesion	217	GO_AMEBOIDAL_TYPE_CELL_MIGRATION	> Cell migration that is accomplished by extension and retraction of a pseudopodium.
		GO_CELLULAR_EXTRAVASATION	> The migration of a leukocyte from the blood vessels into the surrounding tissue.
		GO_IMMUNOLOGICAL_SYNAPSE	> An area of close contact between a lymphocyte (T-, B-, or natural killer cell) and a target cell formed through the clustering of particular signaling and adhesion molecules and their associated membrane rafts on both the lymphocyte and the target cell and facilitating activation of the lymphocyte, transfer of membrane from the target cell to the lymphocyte, and in some situations killing of the target cell through release of secretory granules and/or death-pathway ligand-receptor interaction.
		GO_POSITIVE_REGULATION_OF_BLOOD_VESSEL_ENDOTHE	> Any process that activates or increases the frequency, rate or extent of the migration of the endothelial cells of blood vessels.
		GO_NEGATIVE_REGULATION_OF_CELL_CELL_ADHESION	> Any process that stops, prevents or reduces the rate or extent of cell adhesion to another cell.
		GO_POSITIVE_REGULATION_OF_CELL_CELL_ADHESION	> Any process that activates or increases the rate or extent of cell adhesion to another cell.
		GO_PODOSOME	> An actin-rich adhesion structure characterized by formation upon cell substrate contact and localization at the substrate-attached part of the cell, contain an F-actin-rich core surrounded by a ring structure containing proteins such as vinculin and talin, and have a diameter of 0.5 mm.
		GO_REGULATION_OF_BLOOD_VESSEL_ENDOTHELIAL_CELL_	> Any process that modulates the frequency, rate or extent of the migration of the endothelial cells of blood vessels.
		KEGG_VEGF_SIGNALING_PATHWAY	> VEGF signaling pathway
p38MAPK signaling	84	BIOCARTA_P38MAPK_PATHWAY	> p38 MAPK Signaling Pathway
		KEGG_MAPK_SIGNALING_PATHWAY	> MAPK signaling pathway
		PID_P38_MKK3_6PATHWAY	> p38 MAPK signaling pathway
		PID_P38_MK2_PATHWAY	> p38 signaling mediated by MAPKAP kinases
		ST_P38_MAPK_PATHWAY	> p38 MAPK Pathway
DNA repair	164	DNA_REPAIR	> Genes annotated by the GO term GO:0006281. The process of restoring DNA after damage. Genomes are subject to damage by chemical and physical agents in the environment (e.g. UV and ionizing radiations, chemical mutagens, fungal and bacterial toxins, etc.) and by free radicals or alkylating agents endogenously generated in metabolism. DNA is also damaged because of errors during its replication. A variety of different DNA repair pathways have been reported that include direct reversal, base excision repair, nucleotide excision repair, photoreactivation, bypass, double-strand break repair pathway, and mismatch repair pathway.
		GO_DNA_REPAIR	> The process of restoring DNA after damage. Genomes are subject to damage by chemical and physical agents in the environment (e.g. UV and ionizing radiations, chemical mutagens, fungal and bacterial toxins, etc.) and by free radicals or alkylating agents endogenously generated in metabolism. DNA is also damaged because of errors during its replication. A variety of different DNA repair pathways have been reported that include direct reversal, base excision repair, nucleotide excision repair, photoreactivation, bypass, double-strand break repair pathway, and mismatch repair pathway.
		GO_DNA_REPAIR_COMPLEX	> A protein complex involved in DNA repair processes including direct reversal, base excision repair, nucleotide excision repair, photoreactivation, bypass, double-strand break repair pathway, and mismatch repair pathway.
		GO_DOUBLE_STRAND_BREAK_REPAIR	> The repair of double-strand breaks in DNA via homologous and nonhomologous mechanisms to reform a continuous DNA helix.
		KAUFFMANN_DNA_REPAIR_GENES	> Genes involved in DNA repair, compiled manually by the authors.
		REACTOME_DNA_REPAIR	> Genes involved in DNA Repair
DNA damage	175	BIOCARTA_CHEMICAL_PATHWAY	> Apoptotic Signaling in Response to DNA Damage
		GO_NEGATIVE_REGULATION_OF_RESPONSE_TO_DNA_DAM	> Any process that stops, prevents or reduces the frequency, rate or extent of response to DNA damage stimulus.
		GO_POSITIVE_REGULATION_OF_RESPONSE_TO_DNA_DAM	> Any process that activates or increases the frequency, rate or extent of response to DNA damage stimulus.
		GO_REGULATION_OF_RESPONSE_TO_DNA_DAMAGE_STIM	> Any process that modulates the frequency, rate or extent of response to DNA damage stimulus.
		RESPONSE_TO_DNA_DAMAGE_STIMULUS	> Genes annotated by the GO term GO:0006974. A change in state or activity of a cell or an organism (in terms of movement, secretion, enzyme production, gene expression, etc.) as a result of a stimulus indicating damage to its DNA from environmental insults or errors during metabolism.
		REACTOME_P53_DEPENDENT_G1_DNA_DAMAGE_RESPONS	> Genes involved in p53-Dependent G1 DNA Damage Response
		MODULE_403	> DNA damage response.
p53 signaling	105	HALLMARK_P53_PATHWAY	>Genes involved in p53 pathways and networks.
		BIOCARTA_P53_PATHWAY	> p53 Signaling Pathway
		KEGG_P53_SIGNALING_PATHWAY	> p53 Signaling Pathway
		PID_P53_REGULATION_PATHWAY	> p53 Pathway
Leukocyte migration and adhesion	92	GO_CELLULAR_EXTRAVASATION	> The migration of a leukocyte from the blood vessels into the surrounding tissue.
		GO_LEUKOCYTE_ADHESION_TO_VASCULAR_ENDOTHELIAL_	> The attachment of a leukocyte to vascular endothelial cell via adhesion molecules.
		GO_LEUKOCYTE_CELL_CELL_ADHESION	> The attachment of a leukocyte to another cell via adhesion molecules.
		GO_LEUKOCYTE_MIGRATION	> The movement of a leukocyte within or between different tissues and organs of the body.
		GO_MYELOID_LEUKOCYTE_MIGRATION	> The movement of a myeloid leukocyte within or between different tissues and organs of the body.
		GO_REGULATION_OF_LEUKOCYTE_MIGRATION	> Any process that modulates the frequency, rate, or extent of leukocyte migration.
