# Supplementary material for: Notch1 signaling in NOTCH1-mutated mantle cell lymphoma depends on Delta-Like ligand 4 and is a potential target for specific antibody therapy
Source: J Exp Clin Cancer Res. 2019 Nov 1;38:446. doi: 10.1186/s13046-019-1458-7 (PMC6825347; doi:10.1186/s13046-019-1458-7)
Supplement: Supplementary file 2 — Additional file 2: Table S2. Significantly regulated NOTCH1 target genes upon DLL4 stimulation using a customized NOTCH1 set of genes (NOTCH1 custom) [6, 11, 15, 27–30] [file 13046_2019_1458_MOESM2_ESM.pdf]

**Additional file 2: Table S2**. Significantly regulated *NOTCH1* target genes upon DLL4 stimulation using a customized *NOTCH1* set of genes (NOTCH1 custom)[6, 11, 15, 27-30]

| GENE | DESCRIPTION |
|---|---|
| ABHD6 | abhydrolase domain containing 6 |
| ANGPTL6 | angiopoietin-like 6 |
| BHLHE40 | basic helix-loop-helix family member e40 |
| CCDC138 | coiled-coil domain containing 138 |
| CD27 | CD27 molecule |
| CD7 | CD7 molecule |
| CDC6 | CDC6 cell division cycle 6 homolog (S. cerevisiae) |
| CDK5R1 | cyclin-dependent kinase 5. regulatory subunit 1 (p35) |
| CHEK1 | CHK1 checkpoint homolog (S. pombe) |
| CMTM8 | CKLF-like MARVEL transmembrane domain containing 8 |
| CPPED1 | calcineurin like phosphoesterase domain containing 1 |
| CR2 | complement component (3d/Epstein Barr virus) receptor 2 |
| DOCK5 | dedicator of cytokinesis 5 |
| DTX1 | deltex homolog 1 (Drosophila) |
| ENOX2 | ecto-NOX disulfide-thiol exchanger 2 |
| FYN | FYN oncogene related to SRC. FGR. YES |
| GCET2 | germinal center expressed transcript 2 |
| GINS2 | GINS complex subunit 2 (Psf2 homolog) |
| GUF1 | GUF1 GTPase homolog (S. cerevisiae) |
| HDHD1 | pseudouridine 5'-phosphatase |
| HES4 | hairy and enhancer of split 4 (Drosophila) |
| IRF8 | interferon regulatory factor 8 |
| KCTD9 | potassium channel tetramerisation domain containing 9 |
| LGMN | Legumain |
| LZTFL1 | leucine zipper transcription factor-like 1 |
| MCM10 | MCM10 minichromosome maintenance deficient 10 (S. cerevisiae) |
| MND1 | meiotic nuclear divisions 1 homolog (S. cerevisiae) |
| MYBL2 | v-myb myeloblastosis viral oncogene homolog (avian)-like 2 |
| P2RX1 | purinergic receptor P2X. ligand-gated ion channel. 1 |
| P2RX5 | purinergic receptor P2X. ligand-gated ion channel. 5 |
| P2RY2 | purinergic receptor P2Y. G-protein coupled. 2 |
| PTP4A3 | protein tyrosine phosphatase type IVA. member 3 |
| RCBTB2 | regulator of chromosome condensation (RCC1) and BTB (POZ) domain containing protein 2 |
| RFX8 | RFX family member 8. lacking RFX DNA binding domain |
| SCN2A | sodium voltage-gated channel alpha subunit 2 |
| SEMA7A | semaphorin 7A. GPI membrane anchor (John Milton Hagen blood group) |
| SETBP1 | SET binding protein 1 |
| SFXN1 | sideroflexin 1 |
| SLC1A4 | solute carrier family 1 (glutamate/neutral amino acid transporter). member 4 |
| SLC29A1 | solute carrier family 29 (nucleoside transporters). member 1 |
| TASP1 | taspase. threonine aspartase. 1 |
| TBCD | tubulin-specific chaperone d |
| UCK2 | uridine-cytidine kinase 2 |
| ZFYVE9 | zinc finger. FYVE domain containing 9 |
