# Supplementary material for: Notch1 signaling in NOTCH1-mutated mantle cell lymphoma depends on Delta-Like ligand 4 and is a potential target for specific antibody therapy
Source: J Exp Clin Cancer Res. 2019 Nov 1;38:446. doi: 10.1186/s13046-019-1458-7 (PMC6825347; doi:10.1186/s13046-019-1458-7)

Additional file 3: Table S3: Significantly regulated direct *NOTCH1* target genes upon DLL4 stimulation using a customized set of genes according to Ryan et al. (NOTCH1 direct targets)[26]

MINO CELLS		JEKO-1 CELLS	
ABHD6	abhydrolase domain containing 6	ABHD6	abhydrolase domain containing 6
ARAP1	ArfGAP With RhoGAP Domain, Ankyrin Repeat And PH Domain 1	ARHGAP17	Rho GTPase activating protein 17
B4GALT1	UDP-Gal:betaGlcNAc beta 1,4- galactosyltransferase, polypeptide 1	ARID1B	AT rich interactive domain 1B (SWI1-like)
CAMKK1	calcium/calmodulin-dependent protein kinase kinase 1, alpha	BATF	basic leucine zipper transcription factor, ATF-like
CAMP	cathelicidin antimicrobial peptide	BCL2A1	BCL2-related protein A1
CD27	CD27 molecule	CAMP	cathelicidin antimicrobial peptide
CD84	CD84 molecule	CBFA2T3	core-binding factor, runt domain, alpha subunit 2; translocated to, 3
CDK5R1	cyclin-dependent kinase 5, regulatory subunit 1 (p35)	CD300A	CD300a molecule
CHI3L2	chitinase 3-like 2	CDK5R1	cyclin-dependent kinase 5, regulatory subunit 1 (p35)
COQ2	coenzyme Q2 homolog, prenyltransferase (yeast)	CLEC17A	(C-Type Lectin Domain Containing 17A
CR2	complement component (3d/Epstein Barr virus) receptor 2	CNR2	cannabinoid receptor 2 (macrophage)
CXCR5	C-X-C chemokine receptor type 5	CR2	complement component (3d/Epstein Barr virus) receptor 2
DNASE1L3	deoxyribonuclease I-like 3	CXCR5	C-X-C chemokine receptor type 5
DNASE2	deoxyribonuclease II, lysosomal	CYLD	cylindromatosis (turban tumor syndrome)
ENTPD1	ectonucleoside triphosphate diphosphohydrolase 1	DSE	Dermatan-sulfate epimerase
FCRL2	Fc receptor-like 2	DUSP2	dual specificity phosphatase 2
FCRL3	Fc receptor-like 3	FCRL5	Fc receptor-like 5
FGR	Gardner-Rasheed feline sarcoma viral (v-fgr) oncogene homolog	FLNB	filamin B, beta (actin binding protein 278)
GALNT6	UDP-N-acetyl-alpha-D-galactosamine:polypeptide N-acetylgalactosaminyltransferase 6 (GalNAc-T6)	HES1	hairy and enhancer of split 1, (Drosophila)
HES1	hairy and enhancer of split 1, (Drosophila)	IL10RA	interleukin 10 receptor, alpha
HPCAL1	hippocalcin-like 1	IL16	interleukin 16 (lymphocyte chemoattractant factor)
IFNAR2	interferon (alpha, beta and omega) receptor 2	IL21R	interleukin 21 receptor
IL10RA	interleukin 10 receptor, alpha	IQSEC1	IQ motif and Sec7 domain 1
IRF8	interferon regulatory factor 8	IRF8	interferon regulatory factor 8
KAZN	Kazrin	MYBL2	v-myb myeloblastosis viral oncogene homolog (avian)-like 2
KLF13	Kruppel-like factor 13	NEDD9	neural precursor cell expressed, developmentally down-regulated 9
LGMN	legumain	NEIL2	nei like 2 (E. coli)
LY86	lymphocyte antigen 86	PAX5	paired box gene 5 (B-cell lineage specific activator)
MYBL2	v-myb myeloblastosis viral oncogene homolog (avian)-like 2	PTK2B	PTK2B protein tyrosine kinase 2 beta
NEDD9	neural precursor cell expressed, developmentally down-regulated 9	RAB11FIP4	RAB11 family interacting protein 4 (class II)
NEIL2	nei like 2 (E. coli)	SH2B2	SH2B adaptor protein 2
NRARP	NOTCH Regulated Ankyrin Repeat Protein)	SIK1	Salt Inducible Kinase 1
P2RX5	purinergic receptor P2X, ligand-gated ion channel, 5	SMAD3	SMAD, mothers against DPP homolog 3 (Drosophila)
PAX5	paired box gene 5 (B-cell lineage specific activator)	SPIB	Spi-B transcription factor (Spi-1/PU.1 related)
PLAC8	placenta-specific 8	TET2	Tet Methylcytosine Dioxygenase 2
PRICKLE1	prickle homolog 1 (Drosophila)	TNF	tumor necrosis factor (TNF superfamily, member 2)
PTP4A3	protein tyrosine phosphatase type IVA, member 3	TNFRSF1B	tumor necrosis factor receptor superfamily, member 1B
RAB11FIP4	RAB11 family interacting protein 4 (class II)	UBASH3B	Ubiquitin Associated And SH3 Domain Containing B
RXRA	retinoid X receptor, alpha		
S1PR1	Sphingosine-1-phosphate receptor 1		
SASH3	SAM And SH3 Domain Containing 3		
SEMA7A	semaphorin 7A, GPI membrane anchor (John Milton Hagen blood group)		
SH2B2	SH2B adaptor protein 2		
SH3TC1	SH3 domain and tetratricopeptide repeats 1		
SIK1	Salt Inducible Kinase 1		
SSH2	slingshot homolog 2 (Drosophila)		
ST6GAL1	ST6 beta-galactosamide alpha-2,6-sialyltranferase 1		
SUSD3	sushi domain containing 3		
TBC1D9	TBC1 domain family, member 9 (with GRAM domain)		
TMPRSS3	transmembrane protease, serine 3		
TNFRSF1B	tumor necrosis factor receptor superfamily, member 1B		

Additional file 3. Figure S1: GSEA Enrichment plots upon DLL4 stimulation in Mino and JeKo-1 cells using a customized set of genes according to Ryan et al. (NOTCH1 direct targets)[26]

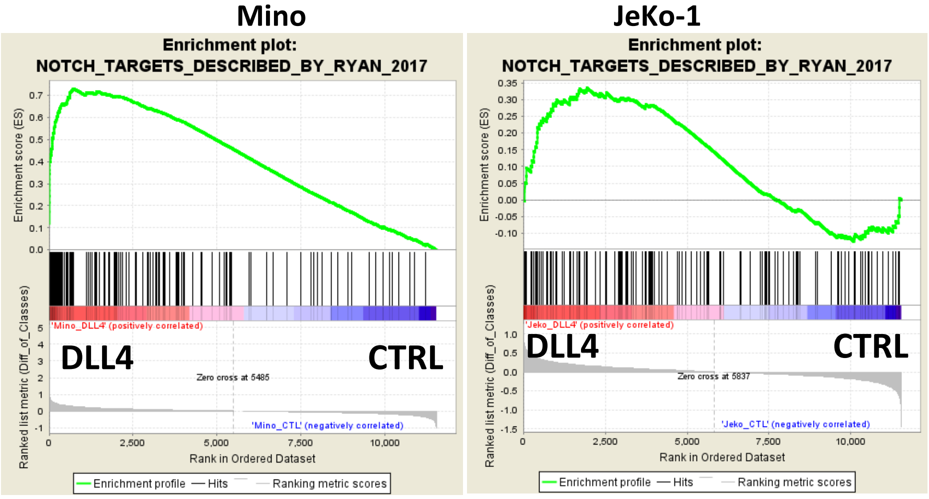

Supplement: Supplementary file 3 — Additional file 3: Table S3. Significantly regulated direct NOTCH1 target genes upon DLL4 stimulation using a customized set of genes according to Ryan et al. (NOTCH1 direct targets) [26]. Figure S1. GSEA Enrichment plots upon DLL4 stimulation in Mino and JeKo-1 cells using a customized set of genes according to Ryan et al. (NOTCH1 direct targets) [26] [file 13046_2019_1458_MOESM3_ESM.pdf]
