# Supplementary material for: Notch1 signaling in NOTCH1-mutated mantle cell lymphoma depends on Delta-Like ligand 4 and is a potential target for specific antibody therapy
Source: J Exp Clin Cancer Res. 2019 Nov 1;38:446. doi: 10.1186/s13046-019-1458-7 (PMC6825347; doi:10.1186/s13046-019-1458-7)
Supplement: Supplementary file 4 — Additional file 4: Table S4. Modulated gene sets upon treatment with OMP-52M51 of DLL4-stimulated Mino cells using a customized set of genes (Custom MCL) [file 13046_2019_1458_MOESM4_ESM.pdf]

Additional file 4: Table S4: Modulated gene sets upon treatment with OMP-52M51 of DLL4-stimulated Mino cells using a customized set of genes (Custom MCL)

Custom gene set name	# Genes in Overlap (k)	# Genes in Gene Set (K)	k/K	p-value	FDR q-value
Cytokine signaling	241	309	0.780	<0.001	<0.001
Migration and Adhesion	176	217	0.811	<0.001	<0.001
Cell Cycle	241	353	0.683	<0.001	<0.001
Apoptosis	92	147	0.626	<0.001	<0.001
DNA repair	120	164	0.732	<0.001	<0.001
NOTCH targets	51	79	0.646	<0.001	<0.001
NOTCH custom	110	154	0.714	<0.001	<0.001
DNA damage	144	175	0.823	<0.001	<0.001
p53 signaling	85	105	0.810	<0.001	<0.001
Leukocyte proliferation	50	92	0.543	<0.001	<0.001
p38MAPK signaling	48	84	0.571	<0.001	<0.001
Angiogenesis	38	54	0.704	<0.001	<0.001
MTOR	11	17	0.647	0.032	0.054
