# Supplementary material for: Notch1 signaling in NOTCH1-mutated mantle cell lymphoma depends on Delta-Like ligand 4 and is a potential target for specific antibody therapy
Source: J Exp Clin Cancer Res. 2019 Nov 1;38:446. doi: 10.1186/s13046-019-1458-7 (PMC6825347; doi:10.1186/s13046-019-1458-7)
Supplement: Supplementary file 5 — Additional file 5: Table S5. Modulated gene sets comparing NOTCH-mutated and wild type lymph nodes from MCL patients using a customized set of genes (Custom MCL) [file 13046_2019_1458_MOESM5_ESM.pdf]

Additional file 5: Table S5: Modulated gene sets comparing *NOTCH*- mutated and- wild type lymph nodes from MCL patients using a customized set of genes (Custom MCL)

Custom gene set name	# Genes in Overlap (k)	# Genes in Gene Set (K)	k/K	p-value	FDR q-value
Cell Cycle	119	353	0.337	<0.001	<0.001
DNA damage	60	175	0.343	<0.001	<0.001
DNA repair	47	164	0.287	<0.001	0.002
NOTCH custom	38	154	0.247	<0.001	0.003
Angiogenesis	22	54	0.407	0.013	0.037
p53 signaling	22	105	0.210	0.006	0.047
NOTCH targets	16	79	0.203	0.014	0.134
Cytokine signaling	42	309	0.136	0.071	0.581
MTOR	6	17	0.353	0.315	0.698
p38MAPK signaling	9	84	0.107	0.314	0.797
Apoptosis	21	147	0.143	0.303	0.802
Leukocyte Migration and Adhesion	13	92	0.141	0.398	0.887
