# Supplementary material for: Notch1 signaling in NOTCH1-mutated mantle cell lymphoma depends on Delta-Like ligand 4 and is a potential target for specific antibody therapy
Source: J Exp Clin Cancer Res. 2019 Nov 1;38:446. doi: 10.1186/s13046-019-1458-7 (PMC6825347; doi:10.1186/s13046-019-1458-7)
Supplement: Supplementary file 6 — Additional file 6: Table S6. Significantly modulated genes (FC=1.75) in lymph nodes from NOTCH-mutated MCL patients [file 13046_2019_1458_MOESM6_ESM.pdf]

Additional file 6: Table S6: Significantly modulated genes (FC=1.75) in lymph nodes from *NOTCH*-mutated MCL patients

	GENE	DESCRIPTION
UPREGULATED	BCAT1	branched chain amino-acid transaminase 1, cytosolic
	BHLHE41	basic helix-loop-helix family, member e41
	CCL18	chemokine (C-C motif) ligand 18
	CHI3L1	chitinase 3-like 1 (cartilage glycoprotein-39)
	CHIT1	chitinase 1 (chitotriosidase)
	CXCL11	chemokine (C-X-C motif) ligand 11
	E2F8	E2F transcription factor 8
	FAM72A	family with sequence similarity 72, member A
	FAM83D	family with sequence similarity 83, member D
	GZMB	granzyme B
	HSD11B1	hydroxysteroid (11-beta) dehydrogenase 1
	KIF14	kinesin family member 14
	KIF23	kinesin family member 23
	MMP12	matrix metallopeptidase 12
	NLGN4X	neuroligin 4, X-linked
	PLAC8	placenta specific 8
	TFEC	transcription factor EC
	TLR8	toll-like receptor 8
	TMPRSS3	transmembrane protease, serine 3
	TRPM4	transient receptor potential cation channel, subfamily M, member 4
	UBE2C	ubiquitin-conjugating enzyme E2C
	UBE2T	ubiquitin conjugating enzyme E2T
DOWNREGULATED	ALOX5	arachidonate 5-lipoxygenase
	IGLJ3	immunoglobulin lambda joining 3
	C1orf132	chromosome 1 open reading frame 132
	CCL21	chemokine (C-C motif) ligand 21
	CSGALNACT1	chondroitin sulfate N-acetylgalactosaminyltransferase 1
	CXCL12	chemokine (C-X-C motif) ligand 12
	IGLC7	immunoglobulin lambda constant 7
	CYAT1	immunoglobulin lambda light chain-like
	DFNA5	deafness, autosomal dominant 5
	FAM129C	family with sequence similarity 129, member C
	FBLN2	fibulin 2
	GPR34	G protein-coupled receptor 34
	GPR82	G protein-coupled receptor 82
	GUSBP11	glucuronidase, beta pseudogene 11
	IGLC1	immunoglobulin lambda constant 1 (Mcg marker)
	IGLL3P	immunoglobulin lambda-like polypeptide 3, pseudogene
	KLRK1	killer cell lectin-like receptor subfamily K, member 1
	MZB1	marginal zone B and B1 cell-specific protein
	NEB	nebulin
	OSBPL10	oxysterol binding protein-like 10
	P2RX5	purinergic receptor P2X, ligand gated ion channel, 5
	P2RY8	purinergic receptor P2Y, G-protein coupled, 8
	PKHD1L1	polycystic kidney and hepatic disease 1 (autosomal recessive)-like 1
	RGS5	regulator of G-protein signaling 5
	SATB1	SATB homeobox 1
	SH3BP4	SH3-domain binding protein 4
	STAP1	signal transducing adaptor family member 1
	TCL1A	T-cell leukemia/lymphoma 1A
	TNFRSF17	tumor necrosis factor receptor superfamily, member 17
	TNFSF8	tumor necrosis factor (ligand) superfamily, member 8
	ZBTB20	zinc finger and BTB domain containing 20
	ZNF404	zinc finger protein 404
	ZNF804A	zinc finger protein 804A
